# Characterization of Three Different Mediterranean Beef Fattening Systems: Performance, Behavior, and Carcass and Meat Quality

**DOI:** 10.3390/ani12151960

**Published:** 2022-08-02

**Authors:** Denise Sánchez, Sònia Marti, Marçal Verdú, Joel González, Maria Font-i-Furnols, Maria Devant

**Affiliations:** 1 IRTA, Ruminant Production, 08140 Caldes de Montbui, Spain; denise.sanchez@irta.cat; 2 Animal Nutrition and Feed Industry, bonÀrea Agrupa, 25210 Guissona, Spain; marsal.verdu@bonarea.com; 3 IRTA, Food Quality and Technology Program, Finca Camps i Armet, 17121 Monells, Spain; joel.gonzalez@irta.cat (J.G.); maria.font@irta.cat (M.F.-i.-F.)

**Keywords:** beef cattle, Angus bulls, growth rate, carcass, crossbred Holstein

## Abstract

**Simple Summary:**

Beef fattening systems present a large diversity according to the effect of the type (genetics and gender) of animals fattened, the nutrition programs, the housing conditions, and days on feed, resulting in different carcass traits and meat qualities. New beef fattening systems are arising in Mediterranean countries raising crossbred Angus bulls seeking new marketing opportunities. One of the strengths of the present study is that all animals of the three compared different production systems, two conventional (crossbred heifers, Holstein bulls) and one innovative (crossbred Angus bulls), were raised following their own commercial program (days on feed, nutrition) under the same housing, care, and weather conditions. Furthermore, the carcass and meat quality parameters were analyzed by using a common methodology. With this experimental design, potential factors like the housing conditions or the methodology used to analyze carcass and meat quality (aging time, cooling temperatures, lab equipment) did not interfere in data interpretation. The results indicated that fattening crossbred Angus bulls is suitable in an intensive fattening program but technical data (performance, meat quality or consumer preferences) do not support it as a better alternative to the current Holstein bull production.

**Abstract:**

The aim of this study was to characterize three different commercial dairy beef fattening systems for intensive Mediterranean fattening programs differing in gender, breed, nutrition, and days of feed in order to describe their performance, behavior, and carcass and meat quality when they were raised simultaneously under the same housing and care conditions. Treatments were three different production systems: (1) crossbred Holstein x beef breeds such as Charolais or Limousine heifers, slaughtered at 10 months of age (CBH10, *n* = 41); (2) Holstein bulls, slaughtered at 11 months of age (HB11, *n* = 42); and (3) crossbred Holstein x Angus bulls, slaughtered at 12 months of age (CAB12, *n* = 37). According to our results, moving from a production system based on Holstein bulls to the crossbred Angus production system has no technical support as no large production and meat quality improvements were observed, and only marketing strategies for meat differentiation and consumer trends could favor this decision.

## 1. Introduction

Beef fattening systems differ widely among European Union countries, with a large diversity in the type (genetics and gender) of animals fattened, in the nutrition programs, in the housing conditions, and in the days on feed, resulting in different carcass traits and meat qualities [1,2]. These beef fattening systems can range from extensive systems in Ireland to very intensive systems in Italy [3,4]. In Spain, the beef fattening system is traditionally an intensive system where calves are housed during the fattening period, commonly in partially open barns with straw bedding [5,6]. Briefly, 30% of the animals fattened are females (mainly crossbred) and from the remaining 70% of males, 30% are Holstein calves [7]. Dairy calves (dairy beef system) account for 40% of the fattened calves, and these calves are fed ad libitum concentrate from very young ages (before the weaning period) [7]. In this dairy beef fattening system, commercially, crossbred females are fattened for shorter periods of time (slaughtered around 10 months of age) to avoid the decrease in feed efficiency [8]. Commonly, these crossbred females are fed the same fattening concentrate with a moderate energy content during the growing and finishing periods [5]. Holstein bulls are usually slaughtered later, before 12 months of age, as the decline of the efficiency with age takes place later compared with the crossbred females. These males are traditionally fed a growing concentrate with moderate energy content until 9–10 months of age and a finishing concentrate with a greater energy content until slaughter [9]. The carcass and meat quality of females and males differ; bulls are sexually more active and more susceptible to suffering pre-slaughter stressors (handling, transportation, lairage) than heifers, and the meat of their carcasses is more prone to become DFD (dark, firm, and dry) [10] than females. In addition, heifer meat has greater intramuscular fat and is more tender than bull meat [11,12]. Recently, in Mediterranean countries, raising crossbred Angus bulls in the dairy beef fattening system has been suggested as a new marketing opportunity. The expectations (hypotheses) are that these crossbred Angus animals would perform close to the Holstein males in terms of growth and close to crossbred females in terms of meat quality [13]. However, there is a lack of data describing crossbred Angus raised in intensive production systems, where calves are fed high-concentrate diets from very young ages as is usual in Mediterranean production systems. Moreover, it is difficult to analyze and compare different production systems as they are the result of a combination of type of animal (breed, gender) and housing, management, nutrition programs (days on feed), and carcass and meat handling procedures [2]. Therefore, one of the strengths of the present study is the comparison of three different beef production systems following their own commercial fattening program (days on feed, nutrition) and raising animals under the same housing, care, and weather conditions; moreover, carcass and meat quality parameters were analyzed with the same methodology. The aim of this study was to characterize three different commercial beef fattening systems in intensive Mediterranean fattening programs differing by gender, breed, nutrition, and days on feed and to describe their performance, behavior, and carcass and meat quality when raised simultaneously under the same housing and care conditions. Data generated from this study are the first step for decision making and offer technical information to consider whether raising crossbred Angus bulls can be a good alternative to Holstein bulls in a Mediterranean dairy beef fattening system.

## 2. Materials and Methods

### 2.1. Experimental Design, Animals, Housing, and Diets

Animals were reared in a commercial farm of Agropecuària Montgai S.L. (Montgai, Spain) and were managed following the principles and guidelines from the Animal Care Committee of the Institute for Research and Technology in Agrifood (IRTA, Caldes de Montbui, Spain). This study was conducted in accordance with the Spanish guidelines for experimental animal protection (Royal Decree 53/2013 of 1 February on the protection of animals used for experimentation or other scientific purposes; Boletín Oficial del Estado, 2013).

The experiment was designed as a randomized, balanced design with covariance adjustment with 3 treatments. Pen was the experimental unit and animals were the sampling units. Treatments were the three different production systems: (1) crossbred Holstein x beef breeds such as Charolais or Limousine heifers, slaughtered at 10 months of age (CBH10); (2) Holstein bulls, slaughtered at 11 months of age (HB11); and (3) crossbred Holstein x Angus bulls, slaughtered at 12 months of age (CAB12). A total of 41 crossbred heifers (CBH10; 165 ± 24.8 kg BW and 141 ± 12.6 d age), 42 Holstein bulls (HB11; 176 ± 18.7 kg BW and 142 ± 4.2 d age) and 37 Angus crossbred bulls (CAB12; 192 ± 52.5 kg BW and 154 ± 34.0 d age) were used to evaluate 3 different production systems and their potential effects on performance, animal behavior, and carcass and meat quality. At arrival, animals belonging to each production system (treatment) were weighed and distributed in pens to equalize initial BW among pens, and then pens were randomly allocated to the 3 treatments (2 pens/treatment; 18–21 animals/pen). Thereafter, animals were weighed at the start of study (d 0) and every 14 d until the end. The CBH10 heifers were slaughtered on d 168 and d 174 of study (BW 425 ± 46.9 kg), HB11 bulls were slaughtered on d 209 and d 216 of study (BW 518 ± 45.7 kg), and CAB12 bulls were slaughtered on d 226 and d 230 of study (BW 554 ± 58.8 kg), simulating commercial criteria in intensive beef fattening in Mediterranean countries.

Animals were allocated in pens (12 m × 6 m) that were equipped with a single-space feeder (0.50 m long × 0.26 m wide × 0.15 m depth) with 10 kg of concentrate capacity, and it was protected by two lateral barriers (1.50 m length × 0.90 m height) forming a chute. Width of chute was regulated from 0.45 to 0.60 m throughout the study to accommodate the increase in animal size and provide sufficient space to allow only one animal to eat comfortably at a time [14], one water bowl (0.30 m length, 0.30 m width, 0.18 m depth) and straw was offered in a separated straw, five-space feeder (3.00 m length, 1.12 m wide, and 0.65 m depth). Animals were offered concentrate, straw, and water ad libitum. Each concentrate feeder was equipped with a scale that consisted of 4 load cells (Ultilcell, Barcelona, Spain), where the feeder was suspended and concentrate contained was continuously weighed, and its weight was displayed by digital screen reader. The scales were calibrated weekly. Every morning, concentrate refusals were recorded as final feed weight of day before; after that, all feeders were automatically refilled via refilling system, and concentrate offers were recorded as initial feed weight of the current day. All feeders were refilled daily by an auger conveying automated feeding distribution system and had a reservoir with storage capacity of 200 kg of concentrate to ensure continuous feed availability, which was dispensed slowly by gravity fall maintaining a continuous and low level of concentrate in the trough. Concentrate intake was considered as concentrate disappearance from feeder, which referred to both concentrate consumption and wastage without discriminating between them because feed spillage was not measured [14]. The amount of straw offered to each pen was recorded weekly to estimate the total amount of straw consumed; however, these data were only an approximation of straw intake because straw was also used for bedding. Before the beginning of the study, animals had a 1-month adaptation period by widening the chute to facilitate feeder access; from then, the width of the chute was adapted to the animal size to allow them to eat easily [15]. Pens were totally covered, i.e., a thick black curtain was installed from the roof to the floor of the barn to avoid eye contact and smelling between heifers and bulls, which could enhance sexual behavior of the bulls. Following commercial feeding practices, heifers (CBH10) were fed the growing concentrate (Table 1) throughout the study, whereas bulls (HB11 and CAB12) were fed the growing concentrate from d 1 to d 168 and the finishing concentrate (Table 1) from d 168 to d 230. Ingredient and nutrient composition from the growing and finishing concentrates (Table 1) were formulated according to FEDNA recommendations [16]. Main differences between the growing and finishing concentrates were metabolizable energy, CP, and fat content. Moreover, animals also had access to barley straw (35 g/kg CP, 16 g/kg EE, 796 g/kg NDF, and 61 g/kg ash; DM basis) and fresh water.

### 2.2. Feed Ingredient Analyses

Feed samples were collected every feed manufacturing for growing and finishing concentrate to analyze dry matter (DM) (method 925.04), ash (method 642.05), crude protein (CP) by the Kjeldahl method (method 988.05) [17], neutral detergent fiber (NDF) [18] using sodium sulfite and alpha-amylase, and ether extract EE by Soxhlet with a previous acid hydrolysis (method 920.39; [17]).

### 2.3. Animal Behavior Evaluation

Animal behavior was recorded for general activities (standing, lying, eating, drinking, and ruminating) and social behavior (non-agonistic, agonistic, and sexual interactions) with a visual scan observation of 2 pens at the same time from 8:00 to 10:00 h [15] for each pen on d 13, 37, 49, 62, 76, 90, 104, 118, 132, 146, 160, 174, 188, 202, and 216, and the last sampling day was 160, 202, or 216 for CBH10, HB11, and CAB12, respectively. General activities were scored using 3 scan samplings of 10 s at 5 min intervals, and social behavior was scored during three continuous sampling periods of 5 min. This scanning procedure of 15 min was repeated twice consecutively in each pen, starting randomly in a different pen every scanning day [15].

### 2.4. Measurements and Sample Collection

Animals from CBH10, HB11, and CAB12 were transported to the slaughterhouse (La Closa, Guissona, Spain) by truck between on d 167 and 174, on d 209 to 216, and on d 226 and 230 of study, respectively, following the EU Regulation 1099/2009 using a captive-bolt pistol and dressed according to commercial practices. Animal transport was organized in six different loads without mixing animals of different treatments and pens. Transport distance was less than 35 km. The hot carcass weight (HCW) was recorded and degree of carcass fatness and conformation were graded according to the EU classification system into 1.2.3.4.5 (EU Regulation No. 1208/81) and into (S)EUROP categories (EU Regulation No. 1208/81, 1026/91), respectively. Dressing percentage was calculated dividing the HCW by the final BW. At 24 h post mortem of carcass chilling at 6.9 °C, pH was measured with a pH meter (pH 25 DL; Crison, Alella, Spain) by penetration of the probe equipped with a xerolyt electrode between the lumbar vertebrae L4 and L5 of the left side of the carcass.

Eighteen samples per treatment (total of 54 carcasses) were selected at random (avoiding the extreme HCW) for meat quality evaluation. At 48 h post mortem, samples from the central part of the *longissimus thoracis* (LT) from rib 12 to 13 were removed from each carcass and cut in 5 steaks of 2.5 cm. Thereafter, three steaks of 2.5 cm were individually packaged in modified atmosphere (MAP; 70% O_2_:30% CO_2_) with polypropylene trays (day 0 of MAP), and the remaining steaks were vacuum-packaged and frozen at −20 °C until determination of intramuscular fat and tenderness.

### 2.5. Meat Analyses

The MAP steaks were displayed for 9 d (11 d post mortem) in an illuminated cooling room (5 ± 0.5 °C) with a homogeneous fluorescent light (900 lx) activated for 12 h a day. From one steak, instrumental color was evaluated on d 2, 6, and 9 post packaging using a Minolta chromameter (CM600d, Minolta Inc., Osaka, Japan) in the CIE-Lab space (L*: lightness, a*: redness, and b*: yellowness; Commission Internationale de l’Éclairage, 1976) with illuminant D65 and 10° viewing angle. In addition, a group of 3 trained panelists evaluated daily a color perception and purchase decision until d 9 post packaging from Monday to Friday. The subjective perception of color and purchase decision were recorded using a 5-point scale (Color perception: (1), highly undesirable; (2), moderately undesirable; (3), slightly desirable; (4), moderately desirable; and (5), highly desirable. Purchase decision: (1), would not buy; (2), would probably not buy; (3), buy dubiously; (4), would probably buy; (5), would buy) [19]. Intramuscular fat was analyzed by using near infrared spectroscopy at wavelengths between 850 and 1048 nm (Foodscan; FOSS, Hillerød, Denmark), previously removing subcutaneous fat and connective tissue and homogenizing with a conventional meat grinder. Instrumental tenderness (Warner–Bratzler shear force (WBSF)) was measured using a texturometer (Stable Micro Systems, Godalming, United Kingdom); samples were thawed for 24 h at 2 °C and wrapped in aluminum foil and baked at 200 °C until the internal temperature reached 71 °C [19]. Cooked steaks were cut into six 1.25 cm diameter cores with a cork borer, parallel to the muscle fiber orientation. The Warner–Bratzer shear blade was perpendicularly oriented to the direction of the fibers [20].

### 2.6. Statistical Analysis

The pen was considered the experimental unit, as the pen was the unit on which all uncontrolled factors were occurring at random. In the case where data were registered individually, the animal was included in the analysis as a sampling unit. A power analysis was conducted to ensure that 2 replicates were appropriate for the statistical power [21].

Initial BW, initial age, final BW, days of study, HCW, and dressing percentage data were analyzed using a mixed-effects model (version 9.4, SAS Inst., Inc., Cary, NC, USA), including treatment as a main effect and pen as a random effect. Initial BW was used as a covariate.

Concentrate intake and average daily gain data were analyzed using a mixed-effect model with repeated measures. The model included initial BW as a covariate, treatment, period, and the interaction between the treatment and period as main effects and pen, animal within pen, and the interaction of treatment and pen as random effects. Period was considered a repeated factor, and the pen nested within treatment was subjected to 2 variance–covariance structures: compound symmetry and autoregressive order. The covariance structure that minimized Schwarz’s Bayesian information criterion was considered the most desirable analysis.

In behavior data, due to the lack of normality of the data analyzed in a previous analysis, the non-parametric Kruskal–Wallis test was performed, and then the data were transformed. Percentage of general activities data were transformed into natural logarithm from behavioral performances, and data from social behavior was transformed into root-square to achieve a normal distribution. These data were analyzed with repeated measures, as described above.

Data from carcass conformation and fatness were analyzed with the FREQ procedure of SAS with the χ^2^ distribution procedure (version 9.4, SAS Inst., Inc., Cary, NC, USA).

Meat quality data, such us pH ultimate, instrumental color and texture at 48 h, and intramuscular fat, were analyzed using a mixed-effects model (version 9.4, SAS Inst., Inc., Cary, NC, USA), including treatment as a main effect and the pen as a random effect. The variables of evolution of instrumental color, color preference, and purchase decision were analyzed using a mixed model with repeated measures as described above, where temperature of the refrigeration room was included as a covariate, treatment day and their interactions were included as fixed effects, and pen, animal within pen, and the interaction of treatment and pen were included as random effects. Least square mean values were compared with Tukey’s HSD test.

Differences were declared significant at *p* < 0.05, and trends were discussed at 0.05 ≤ *p* ≤ 0.10 for all models.

## 3. Results

### 3.1. Performance and Carcass Quality

**Figure 1:**
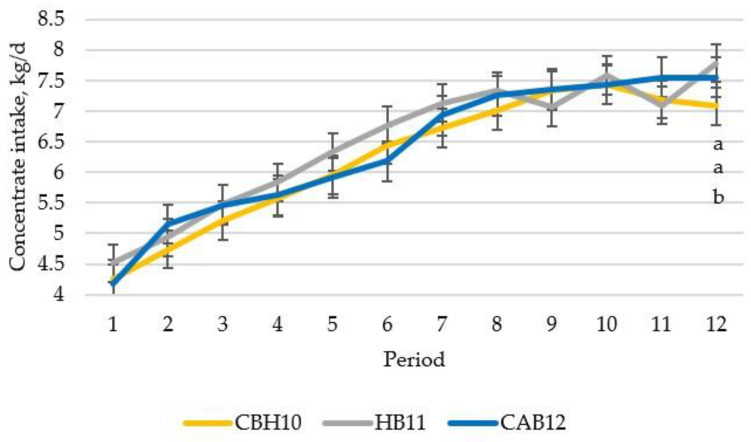
Daily concentrate intake over the time in crossbred Holstein x beef breeds such as Charolais or Limousine heifers, slaughtered at 10 months of age (CBH10); Holstein bulls, slaughtered at 11 months of age (HB11); and crossbred Holstein with Angus bulls, slaughtered at 12 months of age (CAB12) (^a,b^ significant differences (*p* < 0.05) between production system within the same period).

**Figure 2:**
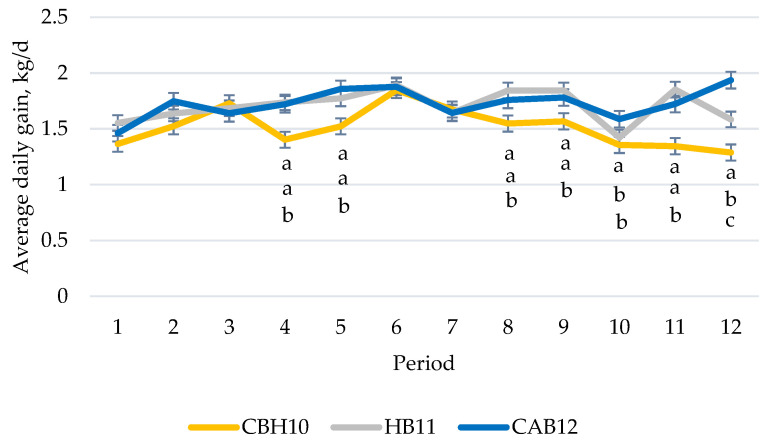
Average daily gain over the time in crossbred Holstein x beef breeds such as Charolais or Limousine heifers, slaughtered at 10 months of age (CBH10); Holstein bulls, slaughtered at 11 months of age (HB11); and crossbred Holstein with Angus bulls, slaughtered at 12 months of age (CAB12) (^a,b,c^ significant differences (*p* < 0.05) between production system within the same period).

Performance and carcass quality data are presented in Table 2. During the first 168 days, total concentrate consumption was greater (*p* < 0.001) only in the last period (Figure 1) for CAB12 and HB11 compared with CBH10 (interaction between production system and period, *p* < 0.001). However, during this 168-day period, ADG of CBH10 were less (*p* < 0.05) than HB11 and CAB12 in most of the periods studied (interaction production system and period was *p* < 0.05) as can be observed in Figure 2. Additionally, as a consequence, feed efficiency data were slightly lower in most of periods studied for CBH10 than for HB11 and CAB12 bulls (interaction production system and period were *p* < 0.001; data not shown). When global performance data were analyzed, as the days in the study differed, differences in total concentrate intake, ADG, and final BW and HCW were observed (Table 2). Total intake of CAB12, despite being slaughtered 16 days later, was close to HB11 (Table 2). Final global efficiency did not differ between both beef fattening systems (Table 2). Moreover, the global efficiency did not differ between raising crossbred heifers and slaughtering them at 10 months of age (CBH10) versus the tested bull production systems (HB11 and CAB12). The HCW of CAB12 slaughtered at 382 d of age was 6.4% greater (*p* < 0.001) than that for HB11 slaughtered at 354 d of age, and the HCW of the HB11 was 14.8% greater (*p* < 0.001) than that for CBH10 slaughtered at 311 d of age. When analyzing the global carcass efficiency (carcass yield expressed by total concentrate consumption) of the fattening bulls, both HB11 or CAB12 were 14% less efficient (*p* < 0.001) than the fattening crossbred heifers slaughtered at 10 months of age. Differences were observed in carcass conformation (*p* < 0.001), resulting CAB12 treatment with the best carcass conformation scoring rate (more “U” and “R” scoring percentages), followed by the carcasses of CBH10 heifers, while the carcasses of the HB11 bulls had the poorest conformation carcasses (greatest percentage of carcasses scored as “P”). However, no differences in the dressing percentage or carcass fatness among the production systems were observed.

### 3.2. Animal Behavior

**Figure 3:**
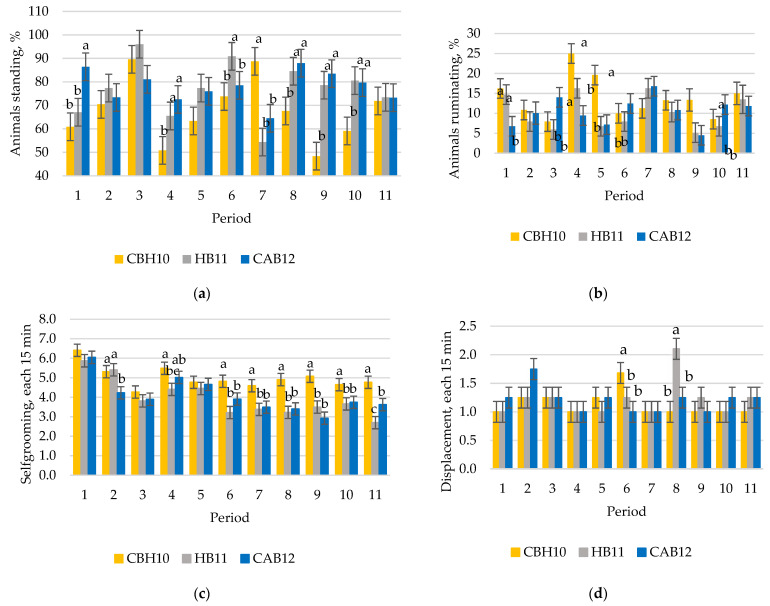
(**a**) Standing, (**b**) ruminating, (**c**) self-grooming, and (**d**) displacement behavior during the first 168 days of study in crossbred Holstein x beef breeds such as Charolais or Limousine heifers, slaughtered at 10 months of age (CBH10); Holstein bulls, slaughtered at 11 months of age; (HB11) and crossbred Holstein with Angus bulls, slaughtered at 12 months of age (CAB12) (^a,b,c^ significant differences (*p* < 0.05) between production system within the same period).

In Table 3, animal behavior data are presented. A significant interaction (*p* < 0.001) between production system and periods was observed in general activities such as standing, lying, and rumination. Although standing and rumination differed among treatments, both behaviors did not follow a regular pattern, as can be observed in Figure 3a–c. Social behavior of CAB12 was less (*p* < 0.05) than CBH10 and HB11 in period 1, HB11 was less (*p* < 0.05) compared with CBH10 and CAB12 in period 4, and CBH10 was greater (*p* < 0.05) compared with HB11 and CAB12 in period 8 (data not represented in figures). Among the agonistic and sexual behaviors, the effect of the production system was more regular. Fighting was less (*p* < 0.01) for CBH10 compared with HB11 and CAB12. Displacement of HB11 was greater (*p* < 0.05) compared with CBH10 and CAB12 in period 8 (Figure 3d). Moreover, HB11 was greater (*p* < 0.01) in chasing compared with CBH10 and CAB12. Flehmen, attempt to mount and complete mount, was less (*p* < 0.001) for CBH10 compared with HB11 and CAB12. Finally, CBH10 performed more stereotyped behaviors (*p* < 0.001) compared with CAB12, and the latter performed more stereotyped behaviors than HB11 (*p* < 0.001).

### 3.3. Meat Quality

**Figure 4:**
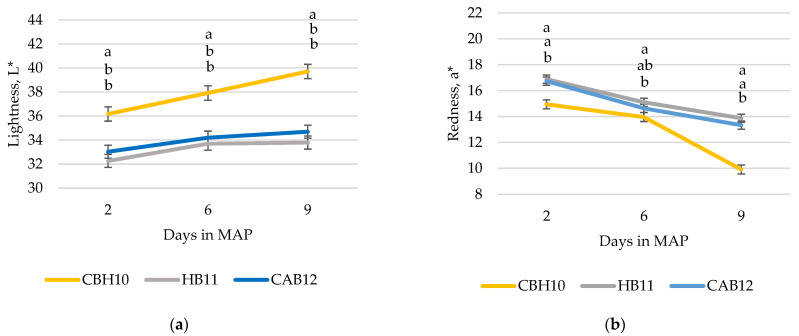

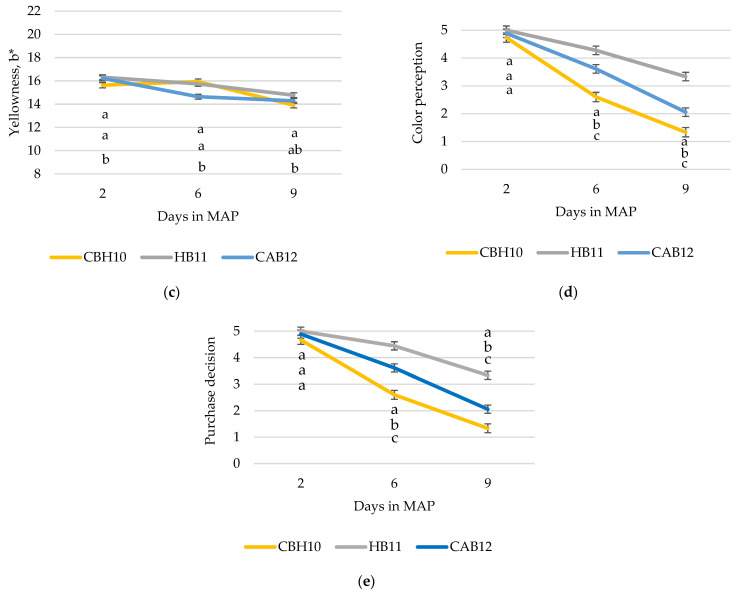
Evolution of the instrumental color ((**a**) lightness, (**b**) redness, (**c**) yellowness)), (**d**) color perception (5-point scale: 1 = highly undesirable, 2 = moderately undesirable, 3 = slightly desirable, 4 = moderately desirable, and 5 = highly desirable), and (**e**) purchase decision (5-point scale: (1) would not buy, (2) would probably not buy, (3) buy dubiously, (4) would probably buy, (5) would buy) of meat over the storage time in refrigeration and MAP conditions by production system (crossbred Holstein x beef breeds such as Charolais or Limousine heifers, slaughtered at 10 months of age (CBH10); Holstein bulls, slaughtered at 11 months of age (HB11); and crossbred Holstein with Angus bulls, slaughtered at 12 months of age (CAB12)) (^a,b,c^ significant differences (*p* < 0.05) between production system within the same day).

Meat pH was greater (*p* < 0.001) for CBH10 and CAB12 compared with HB11 (Table 4). The maximum force and the total area from WBSF were not affected by the production system. Only the slope was significant (*p* = 0.03), but the differences were not relevant. Intramuscular fat had no significant differences between production systems. Lightness (L*) of CBH10 were greater (*p* < 0.001) compared with HB11 and CAB12 during all the conservation time in MAP on days 2, 6, and 9 post-packaging (Figure 4a). Redness (a*) of CBH10 was less (*p* < 0.05) than HB11 and CAB12 at 2 and 9 d, and only less than HB11 at 6 d (Figure 4b). Yellowness (b*) of CBH10 was lower (*p* < 0.05) than HB11 and CAB12 on day 2, higher than CAB12 on day 6, and lower than HB11 at day 9 (Figure 4c). On days 6 and 9, color perception differed among treatments: CBH10 had a less preferred color (*p* < 0.05) than CAB12, which had a less preferred color than HB11. No differences in color perception by meat from different treatments were found at day 2 (Figure 4d). Similar results were obtained by purchase decision (Figure 4e).

## 4. Discussion

When crossbred heifers, Holstein bulls, and Angus–Holstein crossbred bulls were raised under the same housing and management conditions and fed similar diets (concentrate and straw) during the first 168 d of study, heifers, as expected, had a reduced growth and worse efficiency in several growing periods. Some studies have reported that bulls gain weight more rapidly and efficiently than heifers [8], which has been attributed to the anabolic properties of androgens, in particular testosterone [22].

Furthermore, meat from heifers has more intramuscular fat and is more tender than meat from bulls slaughtered at the same age [11,12]; however, in the present study, bulls were slaughtered 41 to 56 days later (Holstein or crossbred Angus, respectively) than heifers, and this could explain the lack of significant differences in intra-muscular fat content since it has been reported that in Holstein bulls intramuscular fat increases around 0.3% every 30 d [21]. It was expected that bulls would be more sexually active and more susceptible to stress than heifers, and they would be more susceptible to pre-slaughter stressors (handling, transportation, lairage), and the meat of their carcasses would be more prone to become DFD (dry, firm, and dark) [10]. However, in the present study, Holstein bulls had the lowest meat pH even if their sexual and agonistic behaviors were not significantly different than those of the crossbred Angus bulls. Some authors have reported differences in pH in bulls slaughtered at different ages and showed that pH was higher at 12 than at 10 and 14 months [21]. Nevertheless, comparisons between works are difficult since the production systems are, not the same, and there are many factors that could affect the pH. The meat pH plays a very important role in technological quality as it largely determines shelf-life and processability as well as water holding capacity and may affect sensory quality attributes, such as the visual perception of color or its tenderness. However, in the present study, differences observed among the three production systems were not considered as relevant since the mean pH was below 6.0 in all production systems. Maybe unrecorded factors such as temperature during transport, waiting time in lairage pens prior to slaughter, or other stressors before slaughtering [23], as well as cooling conditions, could have affected meat quality parameters, such as the pH. In addition, when analyzing the performance data of the global production system, no great differences in efficiency (expressed as growth divided by total concentrate intake) were observed; these results are probably related to the impairment of growth and efficiency with increasing slaughter age [21]. However, a greater impairment of global carcass efficiency (carcass yield expressed as percentage of total concentrate intake) was observed in Holstein or Angus crossbred bulls than in crossbred heifers. Meat quality data, such as intramuscular fat content and meat tenderness, were also very similar among production systems; these data are indicative that each production system had optimized the slaughter age and nutrition program and similar meat quality could be achieved. However, this affirmation is not fully supported by the meat color and purchase decision data. The evolution of the color of the heifer meat (lightness, redness), color perception, and purchase decision were indicative of an unexpected decrease in meat quality and duration of meat shelf-life. These data are not surprising as meat color is the first criterion for consumer appreciation of meat at the time of purchase [24]. Meat color can be affected by many factors (age, gender, type of muscle, intramuscular fat content), but in the present study, the intake of antioxidants via feed may be one of the potential reasons behind this impaired meat color in the heifer meat [25]. The vitamin E content of the growing concentrate was 30 UI per kg, less than the vitamin E content of the finishing concentrate, which was 202 UI per kg; in the present, study animals were fed the same concentrate from day 0 to 168 to be able to compare the different production systems, and the vitamin E concentration was not adapted for the females that were slaughtered at day 170 of the study and, consequently, were not fed finishing concentrate. The authors suggest that supplementing feedlot cattle, especially Holstein steers, with vitamin E extended the color display stability of fresh beef [26]. This was accomplished whether an additional 300 IU/d was supplemented for 9 months, 1140 IU/d for 67 d, or 1200 IU/d for 38 d. In the present study, all animals consumed less than 200 IU daily for 168 days; thereafter, heifers were slaughtered and bulls consumed the finishing concentrate, in which vitamin E was highly increased so that bulls consumed around 1400 IU daily before slaughter, either for 40 or 57 days before slaughter for Holstein bulls or crossbred Angus, respectively. To be able to confirm that vitamin E content was causing the lowest meat color perception scores and the lightness and redness meat, an additional study should be performed. Another unexpected result was that when comparing meat from Holstein bulls vs. crossbred Angus bulls after 6 and 9 days of display in MAP, Holstein bull meat had higher color preference and purchase decision scores than Angus bull meat, although the amount of Vitamin E consumed was higher in the Angus bulls. According to the results of the present study (performance and meat quality data, purchase decision), moving from a production system based on Holstein bulls slaughtered at 11 months to the crossbred Angus bulls slaughtered at 12 months has no technical (performance, animal behavior or meat quality) data support, and only marketing strategies for meat differentiation could support this decision. The decision of crossing dairy cows with Angus was based on the hypothesis that meat from Angus animals is perceived by the consumers as the meat with the best quality [27]; however, the Angus meat that consumers perceive as a good quality meat is usually coming from pure breeds, castrated, probably hormone-implanted animals with a different meat aging protocols, improving meat intramuscular fat, tenderness, and flavor [28]. At the point of purchase, aspects such as color, freshness, appearance, and fat quantity, as well as price and expiration date, are the most important factors for Spanish consumers [29], and, partly, they can be modified with the production system, including breed, age at slaughter, type of feeding, and transport conditions. Thus, any change in the production system should consider all these factors aiming to match consumer demands. As discussed previously, non-technical-based marketing strategies favoring specific claims, such as the production system type, could bias the consumer’s choice, thus pushing the farmers to match market demands.

## 5. Conclusions

No great differences in efficiency (expressed as ADG divided by total concentrate intake), intramuscular fat, or meat tenderness among the three Mediterranean productions systems evaluated (crossbred Holstein x beef breeds such as Charolais or Limousine heifers slaughtered at 10 months of age, Holstein bulls slaughtered at 11 months of age and crossbred Holstein with Angus bulls slaughtered at 12 months of age) were observed. Surprisingly, Holstein bulls had the lowest meat pH even if their sexual and agnostic behavior was not significantly different from the crossbred Angus bulls. However, carcass conformation of crossbred Angus bulls was greater than in Holstein bulls. The evolution of color of heifer meat (lightness, redness), color perception, and purchase decision were indicative of an unexpected impairment in meat quality and meat shelf-life; maybe reduced antioxidant consumption could be one of the potential explanations. Additionally, meat shelf-life was less in crossbred Angus bulls than in Holstein bulls. In summary, according to the present study (performance and meat quality data), moving from a production system based on Holstein bulls to crossbred Angus has no technical support except if carcass conformation wants to be improved, and only marketing strategies for meat differentiation could support this decision.

## Figures and Tables

**Table 1:** Ingredient and nutrient composition of the growing and finishing concentrates.

Item		
Ingredient, g/kg	Growing	Finishing
Corn	421	398
Barley	107	149
Wheat middlings	103	67
Wheat	100	99
Corn DDG	120	99
Peas meal		59
Palm kernel	100	80
Palm oil	10	22
Calcium carbonate	18	14
Urea	8	3
Sodium bicarbonate	4	4
White salt	2	2
Vitamin–mineral premix ^a,b^	3	2
Nutrient, per kg DM		
Metabolizable energy (ME), Mcal/kg	3.18	3.34
CP, g	158	144
Ether extract, g	55	65
Ash, g	54	47
NDF, g	220	198
NFC, g ^c^	511	545

^a^ Premix of the growing concentrate (Montgai, Spain). Vitamins and minerals contained per kg of DM: 3052 kIU of vitamin A, 610 kIU of vitamin D3, 10.2 g of vitamin E, 0.04 g of vitamin K, 10.2 g of vitamin B1, 0.34 g of vitamin B2, 0.04 g of vitamin B6, 0.007 g of vitamin B12, 1.7 g of vitamin B3, 0.2 g of Co, 1.7 g of Cu, 0.2 g of I, 15.3 g of Mn, 0.1 g of Se, 16.7 g of Zn, 200 g of sodium sulfate, 152 g of magnesium oxide, 42.4 g of etoxiquine, 1 kg of barley as excipient. ^b^ Premix of the finishing concentrate (CAG, Guissona, Spain). Vitamins and minerals contained per kg of DM: 3575 kIU of vitamin A, 858 kIU of vitamin D3, 101 g of vitamin E, 2.3 g of vitamin B1, 0.2 g of Co, 2.5 g of Cu, 0.3 g of I, 15.7 g of Mn, 0.2 g of Se, 20.6 g of Zn, 250 g of magnesium oxide, 75.7 g of etoxiquine, 1 kg of barley as excipient. ^c^ NFC = nonfiber carbohydrates calculated as 1000 − (CP + ash + NDF + ether extract).

**Table 2:** Performance and carcass characteristics for the first 168 days and whole study for different Mediterranean beef fattening systems.

	Production System ^1^			SEM ^2^	*p*-Value ^3^		
Item	CBH10	HB11	CAB12		Production System	Time	Production System x Time
Numbers of animals	39	42	37	-	-	-	-
Initial age, days	140	141	154	17.2	0.83	-	-
Initial BW, kg	171	172	188	21.4	0.83	-	-
Performance from 0 to 168 d of study							
Concentrate intake, kg/d	6.24	6.49	6.38	0.138	0.45	<0.001	<0.001
ADG, kg/d	1.53	1.70	1.70	0.030	<0.04	<0.001	0.02
Efficiency, kg/kg	0.25	0.26	0.26	0.010	0.54	<0.001	<0.001
Global performance							
Days of study	171 ^c^	212 ^b^	228 ^a^	0.3	<0.001	-	-
Final age, days	310 ^b^	354 ^ab^	382 ^a^	16.3	<0.01	-	-
Final BW, kg	425 ^c^	523 ^b^	553 ^a^	7.8	<0.001	-	-
ADG, kg/d	1.53 ^b^	1.63 ^a^	1.60 ^ab^	0.027	<0.05		
Total concentrate consumption, kg	1064 ^b^	1437 ^a^	1490 ^a^	22	<0.001		
Efficiency, kg/kg	0.24	0.24	0.24	0.003	0.45		
Carcass parameters							
HCW, kg	243 ^c^	279 ^b^	297 ^a^	4.0	<0.001	-	-
Carcass efficiency, kg/kg	0.22 ^a^	0.19 ^b^	0.19 ^b^	0.003	<0.001		
Dressing percentage, %	55.6	53.7	54.4	0.58	0.17	-	-
Conformation ^4^, %					<0.001		
E	2.6	-	-	-		-	-
U	23.1	-	32.4				
R	33.3	-	67.6				
O	35.9	54.8	-				
P	5.1	45.2	-				
Fatness ^5^,%					1.00		
1	-	-	-	-		-	-
2	2.6	2.4	2.7				
3	97.4	97.6	97.3				

^a,b,c^ Rows with different superscripts differ (*p* < 0.05). ^1^ Treatments CBH10 = crossbred Holstein x beef breeds such as Charolais or Limousine heifers, slaughtered at 10 months of age; HB11= Holstein bulls, slaughtered at 11 months of age; CAB12 = crossbred Holstein with Angus bulls, slaughtered at 12 months of age. ^2^ SEM = standard error of the mean. ^3^ Production system effect; Time = time effect (period of 14 d); Production system x Time = production system by time interaction effect. ^4^ The conformation class designated by the letter “E” (excellent) describes carcasses with all profiles convex to super-convex with exceptional muscle development, and the conformation classified as “U” (very good) describes carcasses with profiles on the whole convex with very good muscle development. The carcasses classified as “R” (good) present profiles, overall, straight and with good muscle development. Carcasses classified as “O” (fair) present profiles straight to concave with average muscle development, and carcasses classified as “P” (poor) present all profiles concave to very concave with poor muscle development. ^5^ The carcass fat cover that is classified as 1 (low) describes none to low fat cover, the class of fat cover classified as 3 (very high) describes an entire carcass covered with fat and with heavy fat deposits in the thoracic cavity.

**Table 3:** Animal behavior for the first 168 days in different Mediterranean beef fattening systems.

	Production System ^1^			SEM ^2^	*p*-Value ^3^		
Item	CBH10	HB11	CAB12		Production System	Period	Production System x Period
General activities, %							
Standing	67.7	76.9	77.9	0.85	<0.001	<0.001	<0.001
Lying	32.3	23.1	22.1	0.85	<0.001	<0.001	<0.001
Concentrate intake	5.0	4.7	5.3	0.05	<0.001	0.22	<0.01
Straw intake	9.7	9.5	12.2	0.89	0.06	<0.001	<0.01
Drinking water	1.1	1.7	1.5	0.28	0.32	0.93	0.48
Ruminating	13.8	10.1	10.5	0.31	<0.001	<0.01	<0.01
Behavior, each 15 min							
Self-grooming	16.6	9.9	10.5	0.20	<0.001	<0.001	<0.001
Social	3.8	2.9	2.7	0.13	0.10	<0.001	<0.001
Oral	5.1	6.4	4.8	0.13	0.22	<0.01	0.52
Fighting	0.7 ^b^	2.9 ^a^	2.1 ^a^	0.21	<0.01	0.29	0.27
Butting	0.4 ^b^	1.3 ^a^	1.3 ^a^	0.14	0.01	0.92	0.50
Displacement	0.2	0.2	0.2	0.05	0.37	0.03	0.03
Chasing	0.1 ^b^	0.6 ^a^	0.1 ^b^	0.09	<0.01	<0.01	0.24
Chasing-up	0.0	0.1	0.0	0.01	0.26	<0.001	0.12
Flehmen	0.1 ^b^	3.3 ^a^	2.7 ^a^	0.16	<0.001	<0.001	0.12
Attempt to mount	0.8 ^b^	3.7 ^a^	2.2 ^a^	0.20	0.03	<0.001	0.15
Complete mount	0.4 ^b^	2.8 ^a^	2.3 ^a^	0.24	<0.01	<0.001	0.98
Stereotype	1.4 ^a^	0.1 ^c^	0.4 ^b^	0.06	<0.001	<0.001	0.05

^a,b,c^ Rows with different superscripts differ (*p* < 0.05). ^1^ Treatments CBH10 = crossbred Holstein x beef breeds such as Charolais or Limousine heifers, slaughtered at 10 months of age; HB11 = Holstein bulls, slaughtered at 11 months of age; CAB12 = crossbred Holstein with Angus bulls, slaughtered at 12 months of age. ^2^ SEM = standard error of the mean. ^3^ Production system effect; Period = time effect (period of 14 d); Production system x Period = production system by period interaction effect.

**Table 4:** Meat pH, shear force, intramuscular fat and evolution of the shelf-life parameters (instrumental color, color perception, and purchase decision) over the time in MAP of meat of animals raised in different Mediterranean beef fattening systems.

	Production System ^1^			SEM ^2^	*p*-Value ^3^		
Item	CBH10	HB11	CAB12		Production System	Days	Production System x Day
pH, 24 h	5.7 ^a^	5.5 ^b^	5.7 ^a^	0.01	<0.001	-	-
WBSF							
Maximum force (kg)	6.6	7.3	6.6	0.38	0.27	-	-
Total area (kg.mm)	69.5	76.9	64.4	5.43	0.27	-	-
Slope (kg.mm)	0.9 ^a^	1.0 ^b^	1.0 ^b^	0.03	0.03	-	-
Intramuscular fat (%)	1.9	1.7	1.7	0.17	0.60	-	-
Instrumental color ^4^							
L*	36.2	32.3	33.0	0.09	<0.001	<0.001	0.03
a*	14.9	16.7	16.9	0.53	<0.001	<0.001	<0.001
b*	15.6	16.2	16.3	0.36	0.03	<0.001	<0.001
Color perception ^5^	2.9	4.2	3.5	0.10	<0.001	<0.001	<0.001
Purchase decision ^6^	2.9	4.3	3.5	0.11	<0.001	<0.001	<0.001

^a,b^ Rows with different superscripts differ (*p* < 0.05). ^1^ Treatments CBH10 = crossbred Holstein x beef breeds such as Charolais or Limousine heifers, slaughtered at 10 months of age; HB11 = Holstein bulls, slaughtered at 11 months of age; CAB12= crossbred Holstein with Angus bulls, slaughtered at 12 months of age. ^2^ SEM = standard error of the mean; ^3^ Production system effect; Day = time effect (2, 6, and 9 d after packaging in MAP); Production system x Day = production system by time interaction effect. ^4^ Instrumental color: L* = lightness, a* = redness, and b* = yellowness. ^5^ 5-point scale, color perception: 1 = highly undesirable, 2 = moderately undesirable, 3 = slightly desirable, 4 = moderately desirable, and 5 = highly desirable. ^6^ 5-point scale, purchase decision: (1) would not buy, (2) would probably not buy, (3) buy dubiously, (4) would probably buy, and (5) would buy.

## Data Availability

Not applicable.
